# Utilizing exosomes as sparking clinical biomarkers and therapeutic response in acute myeloid leukemia

**DOI:** 10.3389/fimmu.2023.1315453

**Published:** 2024-01-16

**Authors:** Wandi Wang, Xiaofang Wu, Jiamian Zheng, Ran Yin, Yangqiu Li, Xiuli Wu, Ling Xu, Zhenyi Jin

**Affiliations:** ^1^ Institute of Hematology, School of Medicine, Jinan University, Guangzhou, China; ^2^ Key Laboratory for Regenerative Medicine of Ministry of Education, Jinan University, Guangzhou, China; ^3^ Department of Pathology, School of Medicine, Jinan University, Guangzhou, China; ^4^ Key Laboratory of Viral Pathogenesis and Infection Prevention and Control, Jinan University, Guangzhou, China

**Keywords:** acute myeloid leukemia, exosomes, prognosis, biomarker, immune evasion

## Abstract

Acute myeloid leukemia (AML) is a malignant clonal tumor originating from immature myeloid hematopoietic cells in the bone marrow with rapid progression and poor prognosis. Therefore, an in-depth exploration of the pathogenesis of AML can provide new ideas for the treatment of AML. In recent years, it has been found that exosomes play an important role in the pathogenesis of AML. Exosomes are membrane-bound extracellular vesicles (EVs) that transfer signaling molecules and have attracted a large amount of attention, which are key mediators of intercellular communication. Extracellular vesicles not only affect AML cells and normal hematopoietic cells but also have an impact on the bone marrow microenvironment and immune escape, thereby promoting the progression of AML and leading to refractory relapse. It is worth noting that exosomes and the various molecules they contain are expected to become the new markers for disease monitoring and prognosis of AML, and may also function as drug carriers and vaccines to enhance the treatment of leukemia. In this review, we mainly summarize to reveal the role of exosomes in AML pathogenesis, which helps us elucidate the application of exosomes in AML diagnosis and treatment.

## Introduction

Acute myeloid leukemia (AML) is an aggressive hematological malignancy that affects adults. It is characterized by the abnormal proliferation and differentiation of immature myeloblasts, which accumulate in the bone marrow (BM) and peripheral blood and impair normal hematopoiesis. Although 50% of patients with AML can achieve complete remission after induction chemotherapy and post-remission treatment, more than 20% of patients with AML remain unresponsive and refractory (1). The precise treatment of AML is impeded by the disease’s aggressive and heterogeneous nature, which is characterized by genetic abnormalities, extensive epigenetic changes, and abnormal tumor microenvironment (TME) (2, 3). Therefore, understanding of the pathogenesis of AML must be improved, and novel biomarkers for diagnosis for its diagnosis and prognosis must be developed.

Exosomes, also known as intraluminal vesicles, are 30-100nm extracellular vesicles (EVs) secreted by various cells, including tumor cells, into the body fluids, blood, urine, semen, saliva, breast milk, amniotic fluid, ascitic fluid, cerebrospinal fluid, and bile (4). The circulatory system serves as the primary medium for exosomes to perform their long-distance communication function (5, 6). Tumor-derived exosomes (TEXs) induce vascular leakage, inflammation, and BM progenitor recruitment during pre-metastatic niche formation and metastasis (7), and finally induce tumor growth and metastasis, affecting tumor progression and prognosis (8).

TEXs in TME transport a substantial amount of genetic material from maternal tumor cells (9). By regulating the physiology of recipient cells, including signaling to tumor and stromal cells, exosomes secreted into the extracellular environment can reshape the TME and promote tumor growth. Exosomes are crucial components in tumorigenesis and tumor proliferation, angiogenesis, invasion, and metastasis (10). This intercellular communication influences cells of various lineages remotely or in situ. Exosomal communication involving immune cells can induce intricate cellular modifications and considerably influence the course of cancer progression by eliciting an immune response. Recent evidence suggests that exosomes greatly influence cell-to-cell and cell-to-environment communication in AML (11). Exosomes are essential for the progression of leukemia and facilitate the survival and chemoresistance of leukemic cells by transferring their molecular cargo (12, 13). Therefore, TEXs are essential to the evaluation of the effects of AML disease activity, severity, and treatment response. In this review, we briefly describe the production of exosomes and how vesicles mediate cellular communication, and then explore the potential use of exosomes in AML diagnosis and treatment.

## Origin and mechanisms of exosomes

Approximately 50 years ago, scientists observed that cells in culture fluid “shed” small vesicles of unknown function -called exosomes. Previously, “waste” produced by cellular physiological metabolism. Owing to the development of high-throughput proteomics and genomics, exosomes have been demonstrated to be involved in intercellular communication in living organisms.

### Exosome biogenesis

Exosome biogenesis is a multistep process involving several pathways. First, multivesicular bodies (MVBs) are generated through two stages of inward membrane budding. The invagination of the cell membrane generates early endosomes, from which exosomes bud inward and late endosomes or MVBs are formed (14). Then, MVBs fuse with the plasma membrane through exocytosis and release exosomes from the cells in tubular vesicles (15). The mechanisms underlying MVB formation including endosomal sorting complex required for transport (ESCRT) pathway, and tetraspanin-dependent pathway (16, 17). ESCRT consists of four subunits (ESCRT-0, ESCRT-I, ESCRT-II, and ESCRT-III) and related molecules (VPS4, VTA1, and ALIX). ESCRT-0 complex initiates the ESCRT pathway through its subunit hepatocyte growth factor-regulated tyrosine kinase substrate, which not only recognizes ubiquitinated proteins and binds to phosphoinositide in the endosomal membrane but also recruits ESCRT-I by binding its TSG101 subunit (18–22). Then, ESCRT-I, and -II promote membrane endosome invagination, ESCRT-III and VPS4 drive the abscission of vesicles from the membrane, and exosomes are generated (23–29). However, the ESCRT system is not the sole pathway for regulating exosome formation. Several tetraspanins, such as CD63, CD81, and CD9, can sequester multiple proteins and form tetraspanin-enriched exosomes (30, 31). Apart from these pathways, other regulators have been identified, including syntenin, ceramide activation via neutral sphingomyelinase, and lipid-raft formation (32–34).

### Secretion of exosome

The mechanism by which MVBs are delivered to the plasma membrane is still not fully understood. Nevertheless, research has demonstrated that the process is controlled by small GTPase molecules that interact with cytoskeletal proteins, and cortactin and ALIX play roles in the intracellular distribution of MVBs (35–37). MVBs fuse with the plasma membrane through a series of proteins, including the soluble N-ethylmaleimide-sensitive factor attachment protein receptor (SNARE). Vesicle-associated membrane proteins bind to the plasma membrane proteins syntaxin and SNAP and then *trans*-SNARE complexes are formed, which provide the necessary force for the movement of MVBs toward the plasma membrane (38). Finally, other SNARE proteins promote the fusion of MVBs and exosome (38–40).

### Exosome uptake

**Figure 1:**
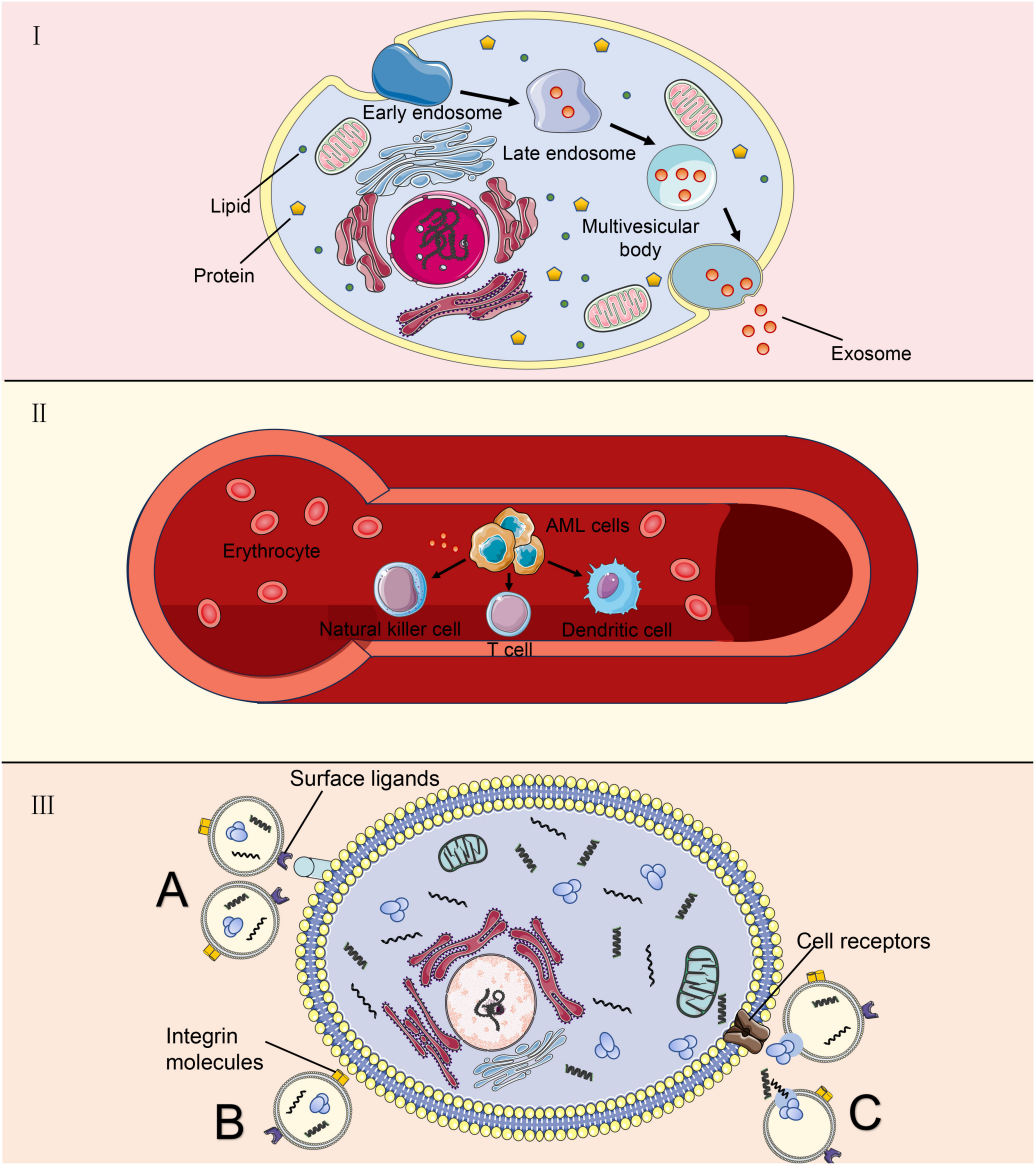
Exosomes derived from AML cells are used as target cells. **(A)** AML cells undergo a process from tubular vesicles (early intracellular bodies) to late intracellular bodies to multivesicular bodies, and finally, they release exosomes into the extracellular space through fusion with the plasma membrane. **(B)** AML-derived exosomes play a long-distance communication role mainly through peripheral blood, which can affect some immune cells; **(C)** Exosomes communicate with target cells. Exosomes can directly activate receptors on the surface of target cells through surface ligands. Integrin molecules on the membrane of exosomal cells directly fuse with the plasma membrane of target cells or endocytosis enters the cell. Outside the cell, exosomes release intracellular substances that bind to receptors on the cell membrane.

After secretion, exosomes with cargoes are released into the extracellular environment. The membranes of exosomes can protect biomacromolecules that exist stably in the body fluid. Therefore, peripheral blood is the main environment in which exosomes perform long-distance communication functions (5, 6). *In vivo*, exosomes can be assimilated by target cells through the direct fusion of membranes, ligand-receptor interactions, or endocytosis. First, exosomes can directly activate receptors on the surfaces of target cells through protein molecules on the surfaces or lipid ligands, generating signaling complexes and activating intracellular signaling pathways (41). Second, in the extracellular matrix, exosomes release intracellular substances that act as ligands to bind to receptors on the cell membrane, thereby activating intracellular signaling pathways (42). Third, exosomes encode essential integrin molecules and fuse with the plasma membranes of target cells or are endocytosed directly into the cells and release nonspecific proteins, noncoding RNA, and nucleic acids (43). Biomolecules transferred by exosomes can alter the phenotypes and functions of recipient cells by altering gene expression and are involved in many physiological and pathological processes in recipient and donor cells (44, 45). **Figure 1** shows the relevant mechanism.

### Content of exosome

Recent data obtained from the Exosome Database reveal that exosomes comprise 1116 lipids, 9769 proteins, 3408 mRNAs, and 2838 microRNAs (miRNAs). The lipid content of exosomes consists of cholesterol, sphingomyelin, ceramide, phosphatidylserine, lysophosphatidic acid, and prostaglandins, which are important for the mechanistic and biophysical aspects of bilayer formation, curvature, and fluidity and affect membrane fusion (46). Proteins in exosomes include tetraspanins, which are membrane transport and fusion proteins on the surfaces of exosomes and act as specific markers. They include specific proteins that are excellent markers for exosome recognition, heat shock proteins (HSP-60, HSP-70, and HSP-90), chaperone proteins, adhesion proteins, MHC (e.g., MHC I and MHC II, which are evolved in antigen presentation), cytoskeletal proteins, multivesicular body synthesis proteins, and lipid-associated proteins (47). In addition, AML-derived exosomes contain the tumor antigens CD33, CD34, and CD117 (48). Exosomes express the adhesion molecules ICAM-1 and integrins, which mediate the interaction and binding of exosomal membranes to receptor cells for cargo delivery (49). Nucleic acids, including DNA, mRNA, and noncoding RNA, are associated with the detection of cancer-associated mutations in serum exosomes (50). Thus, exosome-specific nucleic acids and proteins are crucial for identifying biomarkers of serum exosomes associated with tumor gene mutations and predicting tumor development and prognosis (51).

## AML-derived exosomes cause the dysfunction of immune cells

### Exosomes originating from AML induce T-cell differentiation towards a pro-tumor phenotype

The efficacy of tumor immunotherapy is restricted by tumor cells evading host immune system surveillance and downregulating the function of immune cells, especially antitumor effector cells, including CD8^+^ T and CD4^+^ T cells, natural killer cells (NK), and dendritic cells (DCs) (52, 53). Immune cell dysfunction is a common feature of AML. AML-derived exosomes are key mediators in the TME and function as immunosuppressants, enabling AML cells to evade immune surveillance (12). Exosomes isolated from the plasma of AML patients are loaded with leukemia-associated antigens and inhibitory molecules, which can disrupt the functions of immune cells used in adoptive cell therapy, thereby limiting the expected therapeutic effect of adoptive cell therapy and resulting in immune dysfunction (54). Human TEXs induce apoptosis in activated CD8^+^ T cells, promote the expansion and function of regulatory T (Treg) cells, and thus promote tumor evasion. The proliferation of activated CD8^+^ T cells is inhibited by co-cultivation with TEX, but TEXs increase the proportion of activated CD4^+^ T cells. Additionally, TEXs promote Treg cell expansion and transport transforming growth factor β (TGFβ) and IL-10, which promote the conversion of T cells into Treg cells (55). Treg cells constitute a subpopulation of T cells, mainly CD4^+^CD25^+^ cells, and are classified according to their origin. Elevated levels of Treg cells in peripheral blood are associated with poor outcomes in patients with AML (56). Pando et al. investigated the effects of AML-derived EVs on T cell subsets by an *in vitro* approach to study the effects of EVs derived from the human AML cell line MOLM-14 cells on CD4^+^, CD4^+^CD39^+^, and CD8^+^ T cell subsets from healthy individuals; the results showed that tumor-derived EVs modulate T cell responses by upregulating immune processes, such as immunosuppression and oncogenic gene expression (57).

### AML-derived exosomes downregulate the natural killer receptor of NK cells

NK cells are major innate immune cells in the bloodstream and target tumor cells. In AML, the ability of NK cells to eliminate leukemic cells is dependent on the predominance of activation signals. Weak activation signals among NK cells lead to their inability to exert their cytotoxicity and render them unresponsive to leukemic cells (58). The low expression of the activation receptor natural killer group 2D (NKG2D) of NK cells in patients with AML results in a decline in NK cell activity and the inhibition of its killing function (59). Szczepanski et al. examined serum exosomes from 19 patients with AML and 14 healthy controls and found that serum exosomes from patients with AML disrupted NK cell activation by downregulating the expression of NKG2D; this effect reduced the toxicity of NK cells to tumor cells, but interleukin 15 counteracted this inhibitory effect (60). AML-derived exosomes reduced the cytolytic activity of normal NK cells by downregulating NKG2D receptor expression and inducing Smad phosphorylation in NK cells (61). Hong et al. isolated exosomes from the plasma of the AML human-derived tissue xenograft model they developed; they observed that the expression levels of surface markers in the exosomes were similar to those in the exosomes from patients with AML. The AML-derived exosomes that carried immunosuppressive ligands activated on human NK cell or CD8^+^ T cell receptors, leading to their dysfunction (62).

### AML-derived exosomes inhibit the direct and indirect anti-tumor effects of DCs

**Figure 2:**
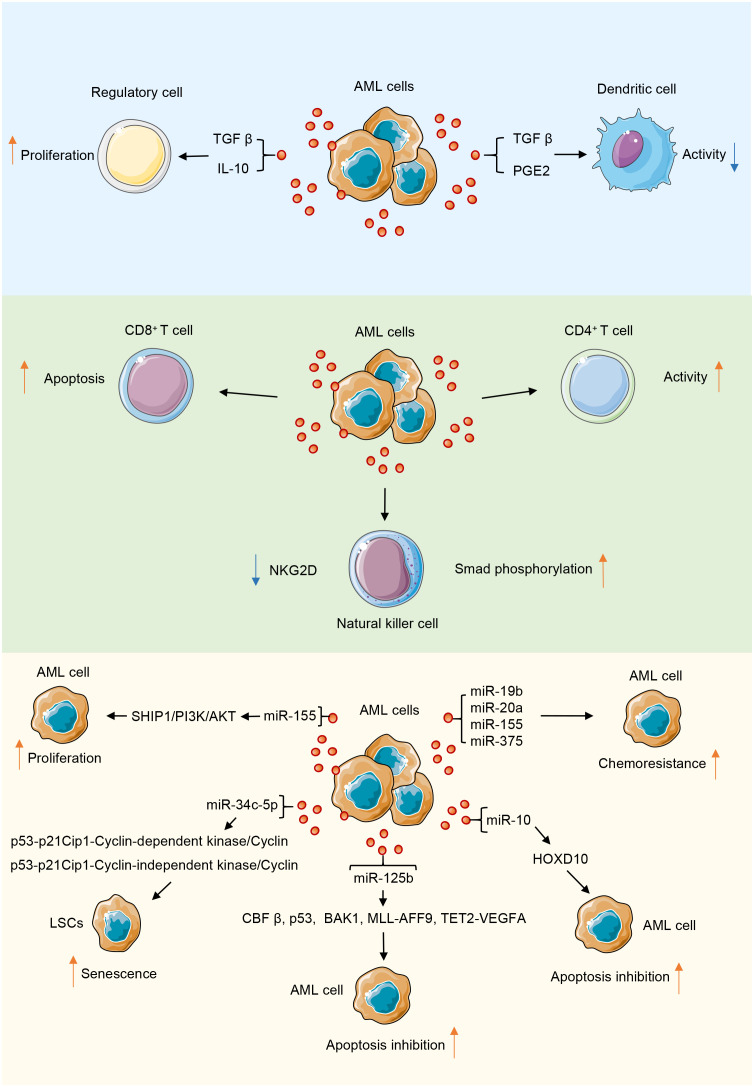
AML-derived exosomes play a role in promoting or inhibiting tumor progression through their contents in the tumor microenvironment. During the interaction of AML-derived exosomes with immune cells, their contents mainly inhibit the function of immune cells.

DCs are major antigen-presenting cells and play an important role in innate immunity. However, DCs generated in the presence of TEX under express costimulatory molecules and produce suppressive cytokines, thus inducing the dose-dependent suppression of T cell proliferation and antitumor cytotoxicity (63). In the context of AML, type I interferons produced by plasmacytoid DCs can clear AML cells. This finding suggests that DCs eliminate AML cells. Benites et al. used exosomes or lysates derived from the leukemia K562 cell line as antigen sources of DC pulses, which initiated the maturation of DCs into a cytotoxic phenotype and markedly enhanced the cleavage of target cells; conversely, when the serum exosomes of patients with AML were used as the pulse sources, opposite effects were observed, which may have induced the immune tolerance of DCs. Considering these contrasting effects can contribute to the mitigation of *in vivo* immune tumor evasion mechanisms (64). In summary, AML-derived exosomes transport substances that induce dysfunction in immune cells and exert a suppressive effect on the immune system (**Figure 2**).

## AML-derived exosomes related to AML progress

### Exosomes participate in BM microenvironment reconstitution

The leukemic microenvironment is a complex and heterogeneous ecological niche composed of various cells, including leukemic, immune, mesenchymal stem, and endothelial cells. The interaction of tumor cells with the microenvironment and tumor stem cells in the BM promotes the relapse of leukemia and metastasis to lymphoid tissues (65). Exosomes are important for the induction of immune responses in a pro-tumor microenvironment and for tumor progression and survival. They promote tumor survival by remodeling the extracellular matrix and inducing angiogenesis and tumor cell proliferation (66). AML can reconstitute the BM microenvironment to one that promotes the growth of leukemic cells but inhibits normal hematopoietic function by secreting exosomes. Exosomes released by AML cells upregulate DKK1 in BM mesenchymal stromal cells and thereby inhibit normal hematopoiesis through the WNT signaling pathway, and AML-derived exosomes stimulate vascular endothelial growth factor (VEGF) signaling in human umbilical vein endothelial cells (HUVECs) by transferring angiogenic factors or proteins and miRNAs, which form vascular tubular structures that promote tumor growth (4, 67). Some studies have confirmed that exosomal miRNAs secreted by AML cells contribute to the progression of AML by altering the expression of downstream genes (68). Point mutation inactivation and reduced SHIP1 gene activity have been observed in patients with AML, and the miR-155-mediated suppression of SHIP1 expression is involved in the pathogenesis of AML. The miR-155/SHIP1/PI3K/AKT signaling pathway potentially has a tumor-suppressive function in the pathogenesis of AML (69). miR-155 is upregulated in FLT3-ITD-associated AML and targets the myeloid transcription factor PU.1. The knockdown of miR-155 inhibits proliferation of FLT3-ITD*-*associated leukemic cells and induces their apoptosis (70). miR-34c-5p is a core miRNA in pathways regulating aging. It is expressed through the p53-p21Cip1-cyclin-dependent kinase (CDK)/cyclin or the p53-independent CDK/cyclin pathway (p53-p21Cip1-CDK/cyclin or p53-independent CDK/cyclin pathway (p53-p21Cip1-CDK/cyclin or p53-independent CDK/cyclin pathways) and promotes leukemia stem cells senescence. However, miR-34c-5p is downregulated in AML (excluding APL) stem cells; poor prognosis and poor therapeutic effect are clinical manifestations of this outcome (71).

### The role of exosomes in the apoptosis of AML

Apoptosis is one of the key mechanisms affecting the survival of AML cells, and the dysregulation of apoptosis may lead to the chemoresistance of AML cells and disease relapse (72). Exosomes carry many complex cargoes, which can serve as the key mediators of intercellular communication and regulate cell proliferation (73). Exosomal miRNAs enter body fluids through autocrine secretion and create a microenvironment in malignant regulatory pathways that facilitate the growth of AML cells by cross talk with other cells, thereby promoting leukemic cell survival, proliferation, and migratory infiltration (74). AML cells highly resistant to apoptosis can affect the expression of apoptosis-related proteins in chemo-sensitive cells. Jiang et al. showed that exosomes secreted by AML cells are enriched in miR-125b (75). The mechanisms by which miR-125b affects apoptosis in AML cells are as follows: First, miR-125b partly targets core binding factor β (CBFβ) and blocks apoptosis by downregulating multiple genes involved in the p53 pathway (76). Second, it inhibits apoptosis and promotes cell proliferation by affecting brassinosteroid-insensitive 1-associated receptor kinase 1 (BAK1) expression (77). Third, miR-125b facilitates the progression of leukemia by promoting the expression of oncogenic MLL-AFF9 *in vivo*, and it upregulates VEGFA, providing conditions conducive to the expansion of leukemic cells. This process involves carcinogenic miRNAs mediating noncellular endogenous leukemia and promoting the miR-125b-TET2-VEGFA pathway. Fourth, caudal-related homeobox transcription factor 2 (CDX2) binds to the promoter region of the miR-125b gene and activates the expression of miR-125b in malignant myeloid cells, and the generated miR-125b inhibits the translation of CBFβ, thereby inhibiting the differentiation of myeloid cells in granulocyte lineaments and promoting the occurrence of leukemia (78). Exosomes in the sera of patients with AML are enriched in miR-10, and miR-10b can inhibit apoptosis and homeobox D10 expression in AML cells by directly targeting homeobox D10 (79).

### Exosomes are involved in the development of drug resistance in AML

Co-culturing of exosomes with multi-drug-resistant AML cell line with chemo-sensitive HL-60 may cause chemo-resistance because of the transfer of miR-19b and miR-20a to the exosomes; thus, exosomes can make chemo-sensitive cells resistant to chemotherapy (80). Chen et al. demonstrated that exosomes secreted by the AML cell KG1α can drive BM stromal cells to produce IL-8, which can inhibit the chemotherapy-induced apoptosis of AML cells (81). Moreover, an exosome-mediated communication mechanism may impede drug therapy. Hekmatirad et al. found that U937 cells (an AML cell line) increase their resistance to the cytotoxic effects of doxorubicin (PLD) in pegylated liposomes through exosome-mediated drug expulsion (82). Another study that investigated the chemoresistance of AML-BMSC exosomes showed that miR-155 and miR-375 in exosomes derived from AML cells are responsible for chemoresistance to chemotherapeutic drugs cytarabine and AC220; the possible mechanism is the miRNA-induced downregulation of the promoters of apoptosis or cell differentiation under the guidance; free leukemic cells become independent of the kinase pathway through this mechanism (83).

## Application of exosomes in the diagnosis and prognosis of AML

Exosomes as biomarkers of tumors have attracted considerable interest (84). They are present in various body fluids and easy to isolate and can be extracted from a small amount of serum (85). Moreover, they have a unique molecular profile (61). AML-secreted exosomal miRNAs are involved in the progression of AML and can be used as entry points for AML treatment (86, 87).

AML might reflect unique miRNA profiles. Compared with the sera of healthy individuals, the sera of patients with tumors contain a large number of exosomes and specific pathogenic information molecules of parental cell origin, which represent the biological behavior of parental cells (88). For example, miRNAs, are important cargoes carried by exosomes because they act in tumor tissues through targeted molecules. miRNAs are important biomarkers of tumor development and prognosis and are protected by exosomal surface membranes with highly conserved sequences. These membranes are stable under extreme conditions and can prevent miRNAs from being released into the circulation (89). miRNAs have potential use in the diagnosis of multiple diseases (90). Exosomal miRNA can be collected from 20 μL of serum and can be used as an ideal molecular marker for the targeted diagnosis and prognosis of leukemia (91). Serum exosomal miR-10b is an independent prognostic factor for overall survival in AML patients. miR-10b expression levels are elevated in the sera of patients with AML, and its expression levels are strongly correlated with poor prognosis, and miR-10b level considerably increases in patients with AML (92–94). Therefore, serum exosomal miR-10b is a potential diagnostic and prognostic marker for AML. The expression levels of miR-146a/b, miR-181a/b/d, miR-130a, miR-663, and miR-135b were high in M1, whereas those of miR-21, miR-193a, and miR-370 were high in M5 (95). In addition, miR-155 is downregulated in peripheral blood mononuclear cells from patients with multi-drug-resistant AML and adriamycin-resistant AML cell lines and showed a positive correlation in these patients. miR-155 can be used as a monitoring indicator for drug resistance and micro-residual focus with high sensitivity (96).

As mentioned above, numerous biomarkers can demonstrate powerful uniqueness in the diagnostic prediction of AML (**Table 1**). AML-derived exosomes are rich in CD33, CD34, and CD117, and their overall protein content is significantly higher than that of healthy controls; the content of some proteins, such as TGF-β1, decreases at the initial diagnosis and effective treatment of AML and can thus be used for detecting leukemia relapse and drug resistance status (97). Plasma exosomal lncRNAs are potential cell-free indicators for the diagnosis and therapeutic monitoring of AML and offer novel and cutting-edge concepts for the liquid biopsy of hematologic cancers (98). Bernardi et al. first used the commercially available CE-IVD-based kits for exosome-enrichment methods to investigate leukemic sources and exosomal dsDNA target resequencing for adult AML marker detection; they performed next-generation sequencing analysis of exosome-derived dsDNA isolated from 14 adult patients with AML and identified the optimal amount of exosomal dsDNA as a potential AML biomarker for liquid biopsies; they found exosomal dsDNA can be developed as a tool that can facilitate the monitoring of AML progression and the early diagnosis of relapse after allogeneic hematopoietic stem cell transplantation (99). In summary, exosomes may offer a novel perspective on AML diagnosis and treatment response (**Table 2**).

**Table 1:** Currently AML biomarkers carried by exosomes.

Substance		Expression	Sample	Reference
Micro-RNA	miR-10b	Upregulated	Bone marrow	(15)
	miR-125b	Upregulated	Plasma	(12)
	miR-155	Upregulated	Plasma	(12)
	miR-21	Upregulated	Bone marrow	(87)
	miR-523	Upregulated	Bone marrow	(88)
	miR-10a-5p	Upregulated	serum	
	miR-93-5p	Upregulated	serum	
	miR-129-5p	Upregulated	serum	
	miR-155-5p	Upregulated	serum	
	miR-181b-5p	Upregulated	serum	
	miR-320d	Upregulated	serum	
Protein	CD33	Upregulated	Plasma	(61)
	CD117	Upregulated	Plasma	
	CD34	Upregulated	Plasma	(47)
	TGF-β1	Upregulated	Plasma	(4)
lncRNA	LINC00265	Downregulated	Plasma	(59)
	LINC00467	Downregulated	Plasma	
	UCA1	Downregulated	Plasma	
	SNHG1	Upregulated	Plasma	

**Table 2:** Promising therapy directions of exosomes in AML.

		Introduction	Reference
Tumor vaccines	TEX-based vaccines	Using exosomes from LEX_TGF-β1si_ as a prophylactic and therapeutic cancer vaccine in a mouse model showed a higher induction-specific antitumor effect, exhibiting more pronounced tumor Growth inhibition and prolongation of survival.	(100)
	T cells-based vaccines	*In vitro* analysis showed that tumor-specific CD4^+^ and CD8^+^ IFN-γ-secreting cells could be efficiently expanded from immunized mice, suggesting that the T helper 1 response is involved in tumor rejection and can kill tumor cells	(82)
	DC-based vaccines	Exosomes are extracted from the serum of AML patients to pulse DC, so that DC recognizes and absorbs the specific antigen contained in it, and DC further activates tumor-specific cytotoxic T cells to generate an immune response and kill AML cells.	(64)
Therapeutic target		Reduce the level of exosome secretion by interfering with the synthesis, release and uptake of exosomes and interfere with their signaling pathways mediated in target cells	(101)
		miR-34c-5p promotes AML cell eradication by selectively targeting RAB27B to inhibit exosome shedding and induce cellular senescence.	(49)
		Transfer of miR-222-3p into THP-1 cells promotes proliferation inhibition and apoptosis of AML cells by targeting the IRF2/INPP4B signaling pathway.	(100)
		miR-29 targets protein kinase b (Akt2) and cyclin D2 proteins, as well as the negative feedback loop of MYC proto-oncogene (c-Myc) -Akt2 on miR-29.	(87)
		miR-451 is involved in the late maturation of erythroid cells, but the introduction of miR-451 into AML cell lines decreased the cell proliferation rate and increased the apoptotic activity.	
Remission of drug resistance		Exosomes can transfer drug resistance between cells through contents, and inhibiting the secretion of these contents helps to alleviate drug resistance	(102)

## Use of exosomes in the treatment of AML

Currently, patients with refractory/recurrent AML experience an aggressive clinical course and have poor prognoses. Therefore, complementary alternative therapies are urgently needed to improve conventional treatments and increase survival rate (103). Blocking exosome-induced production, secretion, and reprogramming has emerged as a novel approach to treating AML and other types of leukemia. This exosome-targeted therapy may be financially beneficial for elderly patients with AML or patients with AML who cannot tolerate strongly induced chemotherapy (101, 104). Therefore, exosome-based immunotherapy has attracted considerable interest. In T-cell lymphoma mice, it effectively eliminated minimal residual disease and prolonged disease-free survival (100). TEX-carrying tumor-associated antigens are potential cell-free tumor treatments for the specific eradication of minimal residual leukemic cells (105). Huang et al. found that lentiviral shRNA– silenced TGF-β1 expression in parental cells of mouse acute lymphoblastic leukemia cell lines and exosomes from TGF-β1-silenced leukemia cells (LEX_TGF-β1si_) had a higher induction-specific antitumor effect than unmodified leukemia cell–derived exosomes. In mouse models, LEX_TGF-β1si_ inhibited tumor growth and prolonged survival as a prophylactic and therapeutic cancer drug (102). This finding suggests that exosomal immune vaccines hold promise for the treatment of leukemia through immunotherapy.

As actionable drug-resistant mediators, exosomes may play a key role in current and future AML treatment. Hekmatirad et al. found that tumor cells can excrete drugs through exosomes and result in resistance and that inhibiting exosome release with GW4869 increases the sensitivity of U937 cells to PLD Therefore, the use of exosome inhibitors is a potential strategy for increasing the sensitivity of cancer cells to treatment. Inhibitors that pharmacologically inhibit exosome release (such as neticonazole, ketotifen, cannabidiol, and GW4869) are effective. Mortality and morbidity in AML are associated with frequent cytopenia, and exosomes may be involved in the suppression of normal hematopoiesis in leukemia (82). Namburi et al. found that exosomes isolated from the plasma of AML patients carry abundant dipeptidyl peptidase 4 (DPP4) and inhibit the differentiation of normal hematopoietic progenitor cells *in vitro*; pharmacologically inhibiting DPP4 reverses exosome-mediated colony formation; therefore, reversing the negative effects of DPP4 exosomes, improving platelet and neutrophil counts, and restoring BM function in patients are promising treatment approaches for AML; however, many regulatory proteins in exosomes contain DPP4 truncation sites and may have different induction, enhancement, or inhibition effects on hematopoiesis (106).

The systematic design of drug delivery vehicles can address many issues, such as low water solubility, poor biocompatibility, rapid metabolism *in vivo*, easy accumulation in nonpathological tissues, and poor ability to penetrate the membranes of some drugs (107). Exosomes as drug delivery vehicles are mainly dependent on their unique natural physicochemical properties, including phospholipid membrane structure that protects them from destruction by nucleases and proteases, high stability in blood, and long blood half-life (108). Nanoscale and lipid bilayer membranes prevents their removal by mononuclear phagocytes and reduce immunogenicity (especially of autologous cell origin), resulting in low cytotoxicity. The specific lipid and protein composition makes them highly stable in body fluids and enables them to readily fuse with target cells, rendering them chemotactic for specific target cells. Exosomes possess distinctive membrane structures that enables them to easily cross the biofilm barrier and act as carriers through specific delivery modes and modifications (109). These advantageous features of exosomes as drug carriers render them highly attractive for precision medicine. Bellavia et al. used HEK293T cells to express Lamp2b, an exosomal protein fused to an IL-3 fragment, and showed that IL3-Lamp2b exosomes loaded with imatinib targeted chronic myelogenous leukemic cells and inhibited cancer cell growth *in vitro* and *in vivo* (110). Kim et al. used exosomes to deliver paclitaxel or doxorubicin to mitigate multidrug resistance in lung cancer (111).

However, exosomes as drug carriers for AML treatment are currently underdeveloped.

## Conclusion

During the progression of AML, exosomes secreted by AML cells can promote the development of AML by affecting the proliferation and apoptosis of AML cells, regulating the BM microenvironment, affecting angiogenesis, and inhibiting hematopoiesis. Therefore, according to the characteristics of AML cell-derived exosomes, exosomes can also be used as biomarkers of AML prognosis, preparing vaccines and drug carriers.

## Author contributions

WDW: Writing – original draft. XFW: Writing – original draft. RY: Visualization, Writing – review & editing. JMZ: Validation, Writing – review & editing. YQL: Writing – review & editing. XLW: Writing – review & editing. LX: Writing – review & editing. ZYJ: Writing – review & editing.
